# The Preparation of Curcumin-Loaded Pickering Emulsion Using Gelatin–Chitosan Colloidal Particles as Emulsifier for Possible Application as a Bio-Inspired Cosmetic Formulation

**DOI:** 10.3390/pharmaceutics16030356

**Published:** 2024-03-03

**Authors:** Beena G. Singh, Nalin Bagora, Minati Nayak, Juby K. Ajish, Nitish Gupta, Amit Kunwar

**Affiliations:** 1 Radiation and Photochemistry Division, Bhabha Atomic Research Centre, Trombay, Mumbai 400085, India; beenam@barc.gov.in (B.G.S.); nminati@barc.gov.in (M.N.); kuttan@barc.gov.in (J.K.A.); 2 Homi Bhabha National Institute, Anushaktinagar, Mumbai 400094, India; 3 Department of Applied Chemistry, S. G. S. Institute of Technology and Science, Indore 452003, India; bagoranalin1995@gmail.com (N.B.); nitish.nidhi75@gmail.com (N.G.)

**Keywords:** colloidal particles, cellular uptake, green formulation, polyelectrolyte, rheology, sun protection factor

## Abstract

In the field of preparing cosmetic formulations, recent advances recommend the usage of excipients derived from biocompatible materials. In this context, the present study aimed to prepare and characterize the curcumin-loaded Pickering emulsion for possible applications in cosmetic formulation. The coconut oil which is often the component of skin care formulations is used as the oily phase. Curcumin, which is well known for absorbing solar radiation, is expected to work synergistically with coconut oil towards improving the sun protection factor (SPF) of the formulation. Additionally, curcumin can also protect the intracellular components through its well-known antioxidant mechanisms. The Pickering emulsion of coconut oil into water was prepared using the composite colloidal particles derived from β-carboxymethyl chitosan (CMC) and Gelatin-A (GA) as the emulsifying agent. The reaction conditions in terms of the weight ratios of CMC and GA, the pH of the reaction medium, the oil volume fraction, and the homogenization speed were optimized to obtain the most stable Pickering emulsion. The obtained systems were physico-chemically characterized by dynamic light scattering, zeta potential, optical microscopy, and rheometric measurements. The final CMC-GA-stabilized emulsion demonstrated an oil droplet size of 100 µm and a SPF_spectrophotometric (290–320 nm)_ value of 8.5 at a curcumin loading of 4 mg/mL. Additionally, the final formulation facilitated the uptake of curcumin into fibroblast (WI26) cells under in vitro conditions. Together, the investigation demonstrates a bio-inspired approach to prepare a curcumin-loaded green Pickering emulsion using biocompatible pharmaceutical grade excipients, which may find utility in cosmetic applications.

## 1. Introduction

Turmeric, or Curcuma longa extract, has traditionally been used in skin care formulations as a beautifying and anti-aging agent [1]. Recent reports suggest that curcumin, the most active constituent present in turmeric extract, protects skin cells from solar UV rays and sunburn via the antioxidant and anti-inflammatory mechanisms [2,3]. Briefly, solar UV rays generate reactive oxygen species (ROS) within skin cells, which, in turn, cause damage to DNA, proteins, and lipids, and activates pro-inflammatory transcription factors (like nuclear factor kappa B, among others), finally leading to phototoxicity and inflammatory responses [2,3]. Curcumin not only directly scavenges ROS, but also induces cellular antioxidant defense mechanisms through nuclear factor erythroid 2-related factor 2 (Nrf2)-dependent pathways [2,3]. Furthermore, curcumin also suppresses solar UV ray-induced inflammatory responses by inhibiting the activation of NF-κB and other pro-inflammatory transcription factors [2,3]. Moreover, curcumin is reported for a wide range of medicinal properties, and is also very well tolerated by the human body, even up to 8 g a day, without showing any significant toxicity symptoms [4,5,6,7]. All these facts have prompted researchers to develop curcumin-based cosmetic formulations, such as sun-screening cream or for general skin care applications [8,9]. As skin cosmetics and/or the sun-screening creams are generally water-based formulations, the major challenge in developing curcumin-based cosmetics is its low aqueous solubility. This results in the poor loading of curcumin into cosmetic formulations. In this context, emulsions have emerged as the most convenient way of formulating antioxidants like curcumin and other bioactive compounds for various industrial and pharmaceutical applications [10,11,12]. Emulsions are a type of colloidal systems that are formed by mixing two immiscible liquids. In general, the thermodynamically unstable emulsions are stabilized using emulsifiers that are surface active agents. The classical emulsions have certain intrinsic limitations, such as instability. With recent advancements in technology, the limitation can be addressed by using solid particles as emulsifiers [13]. Ramsden in 1903 and Pickering in 1907 [14,15] studied such emulsions for the first time, which were later named Pickering emulsions. They can be either water-in-oil (W/O), oil-in-water (O/W), or even multiple emulsions [16,17]. The basic difference between the conventional and the Pickering emulsion is that, in the case of conventional emulsions, the oil droplets in water are stabilized due to the amphiphilic nature of the emulsifier, which is generally the surfactant. Whereas, in the case of the Pickering emulsion, the solid particles of the emulsifier should not be amphiphilic. Instead, solid particles, depending on their size, stably dock at the space between the oily and water phases. Here, the particles of submicron sizes can stabilize the micromillimeter-to-millimeter size of the oil droplets, and this is the greatest advantage of the Pickering emulsion when compared to the conventional emulsion. Furthermore, Pickering emulsions are extremely stable during coalescence, and can facilitate superior stability, low toxicity, and stimuli-responsiveness compared to the classical emulsions. Both inorganic and organic particles can effectively serve as Pickering emulsifiers. Although the surfactant-free characteristics of Pickering emulsions make them favorable for applications in cosmetic formulations, there is a growing concern among the consumers regarding the safety of the ingredients or chemicals used as emulsifiers or solid particles for the preparation of Pickering emulsions [18]. Accordingly, finding an appropriate, safe, sustainable, and effective bio-based natural emulsifier must be prioritized. Recently, a select few bio-compatible macromolecules, like chitosan aggregates, cellulose nanocrystals, egg granules, and starch granules, have been explored as green emulsifiers [19,20,21]. Compared with other biopolymers, protein particles are better emulsifiers due to the fact that the hydrophilicity of their surfaces can be tuned via modulating electrostatic charge. Furthermore, proteins create soft particles that are more conducive for compact packaging at the interface, allowing for the stabilization of the Pickering emulsion [22]. The protein particles are mainly prepared via heat induction and anti-solvent precipitation methods. Liu et al. prepared soybean protein nanoparticles using a heat-induced method, and the internal structure of the nanoparticles was mainly maintained through hydrophobic interactions and disulphide bonding [23]. Folter et al. found that the physico-chemical properties of zein colloidal particles prepared via an anti-solvent precipitation procedure are influenced by the precursor concentration, pH and ionic strength [24]. The zein colloidal particles demonstrating the capacity to stabilize O/W Pickering emulsions. Besides the plant proteins, animal proteins are also being studied. The whey protein microgel particles are new food-grade particles that can stabilize Pickering emulsions, and the adsorption efficiency of these particles strongly depends on the particle charge or the ionic strength of the aqueous phase [24]. Gelatin is another abundantly available animal protein, which is an FDA-approved excipient for pharmaceutical applications. It is a hydrolyzed product of collagen resulting from the action of acid, alkali, enzyme, or high temperatures. This mainly comes from animal skin, bones, tendons, and other by-products of animal food processing [25]. Gelatin is suitable for the preparation of soft Pickering microparticles, due to its gelling properties. However, the preparation of gelatin nanoparticles to stabilize the Pickering emulsion is very difficult, owing to its strong hydrophilicity and thermal dissolution [26]. Therefore, there are few reports on gelatin nanoparticles and their application in the stabilization of Pickering emulsions. Tan et al. [27,28] reported that the gelatin nanoparticles can be prepared using glutaraldehyde as a cross-linking agent and using acetone as an anti-solvent. However, the use of acetone and glutaraldehyde restrict their usage for any green applications.

Therefore, the objective of the present investigation is to design a green Pickering emulsion to be used as a carrier for curcumin, and to explore its potential for cosmetic application. For this, β-carboxymethyl chitosan (CMC) and gelatin-A (GA) were chosen as emulsifiers, due to these ingredients being bio-sourced polyelectrolytes which can interact and form complex or colloidal particles [29]. Furthermore, coconut oil is naturally used for skin care and was therefore chosen for the oily phase [30]. The electrostatic interactions of CMC with GA were examined in detail in order to optimize the conditions of the formation of composite green colloidal particles. Subsequently, CMC-GA composite particles were used as an emulsifier to prepare (coconut) oil-in-water-based Pickering emulsions. Finally, the emulsion was loaded with curcumin and studied for its sun protection factor (SPF) and the cellular release of curcumin using a model human lung fibroblast (WI26) cell. To the best of our knowledge, this is the first report investigating the loading of curcumin into the CMC-GA particle-stabilized Pickering emulsion and its potential for cosmetic applications.

## 2. Materials and Methods

### 2.1. Reagents

GA (bloom strength 175) was purchased from Alfa Aesar (Haverhill, MA, USA). CMC was gifted by Shri S.P. Ramnani (Ex-RTDD, BARC). The coconut oil was purchased from Parachute (Marico Ltd., Mumbai, India). The obtained GA and CMC were characterized by physicochemical properties, such as the isoelectric point (IEP) and the degree of deacetylation (Figure S1A–C), as per the methods mentioned in the reference [31,32]. Curcumin and all other chemicals were purchased from Sigma-Aldrich (St. Louis, MI, USA). The Bradford assay kit was obtained from GeNei, Bangalore, India. Dulbecco’s Modified Eagle Medium (DMEM), Fetal Bovine Serum (FBS), penicillin, and streptomycin were obtained from HiMedia Laboratories Pvt Ltd., Thane, India. All the studies were carried out in triplicates, and the results are presented as mean ± SD.

### 2.2. Preparation and Characterization of CMC-GA Complexes 

The reaction conditions for the formation of CMC-GA colloidal particles were optimized for subsequent applications as the emulsifier for the preparation of the Pickering emulsion. Since the size and surface charge of insoluble particles are important criteria for their applicability as an emulsifier, CMC-GA colloidal particles were characterized via measuring their hydrodynamic size, turbidity, and zeta potential. Briefly, CMC was dissolved in deionized water, and stirred with a magnetic stirrer at 300 rpm for 4 h, then stored overnight at room temperature. Similarly, GA was dissolved in deionized water at 40 °C, and stirred at 300 rpm for 30 min. The CMC-GA mixtures were prepared through mixing CMC and GA solutions at a volume ratio of 1:1, 1:2, 2:1, and 1:4 (CMC:GA), and stirred at 300 rpm for 30 min at room temperature. The mixture was used for the characterization of the hydrodynamic size and surface charge to optimize the concentration the of CMC and GA required for their interaction. Subsequently, the effect of the pH level on the formation and growth of CMC-GA colloidal particles was studied through the measurements of the turbidity and the hydrodynamic size of the reaction mixture for up to 15 days. The pH of the CMC-GA reaction mixture was adjusted using NaOH or HCl solutions. The hydrodynamic sizes of the CMC-GA particles were determined using a dynamic light scattering (DLS) technique on a Malvern 4800 (UK) Autosizer which employed a 7132-digital correlator. The He-Ne laser operated at 632.8 nm with a power output of 15 mW and was used as the light source. Measurements were made at a 130° scattering angle. The turbidity, arising mainly from the change in the size of the complex particles in the solution, is a very important indicator of colloidal particles [33]. The turbidity profiles of the CMC-GA complexes were examined spectrophotometrically (JASCO V-630, JASCO Corporation, Tokyo, Japan) using a quartz cuvette with a 1 cm path length. The spectral measurements were taken in transmittance mode (% T) at 700 nm, and the turbidity index (100 − % T at 700 nm) was calculated. The surface charge of the colloidal particles was determined via the zeta potential measurements (Zetasizer Nano, Malvern, UK), operated at voltages of up to 200 V.

### 2.3. Preparation of Emulsion

Briefly, the CMC-GA mixture was prepared at a pH level of 7 as described above, aged for 3 days in order to allow the formation of insoluble complexes, mixed with coconut oil at an oil volume fraction (φ) varying from 0.1 to 0.7, and then homogenized at varying speeds (2500–16,000 RPM) using a high-speed homogenizer (Kinematica Polytron PT 3100 D, Fisher Scientific, Newington, NH, USA). All the emulsions were stored at room temperature and evaluated for stability by measuring the creaming index, oil droplet size, and rheometric parameters. The methodologies for the determination of these measurements are given in the following sections. 

### 2.4. Determination of Creaming Index

The freshly prepared CMC-GA-stabilized emulsions were kept at room temperature for 30 days, and observed visually for the phase separation (cream and serum). The height of the cream and serum phases were measured using the standard 15 cm scale. The creaming index (*CI*) was calculated using Equation (1), as reported in the previous literature [34].

(1) $$CI=\frac{\mathrm{The}\;\mathrm{height}\;\mathrm{of}\;\mathrm{the}\;\mathrm{serum}\;\mathrm{layer}}{\mathrm{Total}\;\mathrm{height}\;\mathrm{of}\;\mathrm{the}\;\mathrm{emulsion}}\times 100$$

### 2.5. Optical Microscopy of Emulsion

For optical microscopy, the emulsion (10 µL) was spread on the glass slide, mounted with a cover slip, and imaged using the Olympus IX-83 fluorescence microscope (Olympus, Tokyo, Japan).

### 2.6. Rheometric Measurements

The rheometric measurements of the emulsions were carried out using cylindrical plate geometry (Anton Paar Physica MCR101, PP25-4, Graz, Austria) at 25 °C to determine certain parameters, such as the storage moduli (G′), loss moduli (G″), and loss factor or tan (δ). All the dynamic measurements were performed in the linear viscoelastic region, which was determined at a 1% strain amplitude. The viscoelastic properties were measured in a 0.1–100 rad/s frequency range.

### 2.7. Curcumin Loading in the Emulsion

A specified weight of curcumin (2 mg/mL and 4 mg/mL) was dissolved in the oil, and was then warmed at 40 °C until complete dissolution. This solution was then homogenized with the CMC-GA aqueous colloidal suspension at a speed of 16,000 rpm, followed by centrifugation at 13,000 rpm for 10 min to remove the excess undissolved curcumin. Subsequently, 1 μL of the resulting emulsion was diluted in 3 mL of the acetonitrile solvent, and the amount of curcumin was estimated using the extinction coefficient of 6.1 × 10^4^ M^−1^ cm^−1^ at 420 nm. The loading capacity was estimated using the following equation:

(2) $$\begin{array}{l} \mathrm{Encapsulation}\;\mathrm{Efficiency}=\frac{\mathrm{Amount}\;\mathrm{of}\;\mathrm{Curcumin}\;\mathrm{loaded}\;}{\mathrm{Total}\;\mathrm{amount}\;\mathrm{of}\;\mathrm{Curcumin}}\times 100 \end{array}$$

The loading of curcumin into the emulsion was also confirmed through fluorescence microscopy. Briefly, the curcumin loaded-emulsion (10 µL) was spread on the glass slide, mounted with a cover slip, and imaged using the Olympus IX-83 fluorescence microscope (Olympus, Tokyo, Japan).

### 2.8. Determination of the Sun Protection Factor (SPF)

The efficacy of a sunscreen is usually expressed by the SPF [35,36], which is defined as the UV energy required to produce a minimal erythema dose (MED) on the protected skin, divided by the UV energy required to produce an MED on unprotected skin. The MED is defined as the lowest time interval or dosage of UV light irradiation required to produce a minimal, perceptible erythema on unprotected skin. The SPF of the emulsion was determined based on the spectrophotometric analysis of dilute solutions (1% *v*/*v* of final formulation), as reported in the literature [35]. Briefly, 10 μL of the curcumin-loaded emulsion was diluted into 1 mL of ethanol. The absorbance of the resulting solution was recorded in the wavelength region of 290 nm to 320 nm at an increment of 5 nm, and the SPF was calculated using the following Equation (3):

SPF_spectrophotometric_ = CF × EE (λ) × I (λ) × Abs (λ) (3)

Here, EE—Erythemal effect spectrum, I—Solar intensity spectrum, Abs—Absorbance of sunscreen product at 290–320 nm, and CF—correction factor. The value of the CF was taken as 10. Similarly, the value of the EE (290–320 nm) × I (290–320 nm) was taken as 1 from the previous references [35,36].

### 2.9. Cellular Uptake Studies

The human lung fibroblast cells (WI26) obtained from the National Centre for Cell Science (Pune) were maintained as per the technical sheet of ATCC. The cellular uptake of curcumin in the cells was detected as mentioned in our previous reports [37]. Briefly, for fluorescence imaging, the cells were grown on the glass cover slips, treated with the curcumin-loaded coconut oil–water emulsion for 6 h, washed thrice with the cold phosphate-buffered saline to remove the membrane-bound curcumin, fixed with 4% paraformaldehyde, and then imaged using the Olympus IX-83 fluorescence microscope using a band pass fluorescence filter set of FITC. For the quantitative estimation, 5 × 10^6^ cells were treated with the curcumin-loaded coconut oil–water emulsion for 6 h at a curcumin equivalent concentration of 25 µM, washed thrice with the cold phosphate-buffered saline to remove the membrane-bound curcumin, lysed with water containing 0.1% triton X-100, and monitored for absorbance at 420 nm using a multi-well plate reader (Bio Tek Synergy H1 microplate reader, Agilent Technologies, k California, USA). The protein content in the cell lysate was estimated using the Bradford assay kit. The amount of curcumin in the lysate was determined using the calibration curve of the curcumin prepared in the lysis buffer, and normalized with respect to the protein content of the cell.

## 3. Results

### 3.1. Preparation of Composite Colloidal Particles through the Interaction between CMC and GA

**Figure 1:**
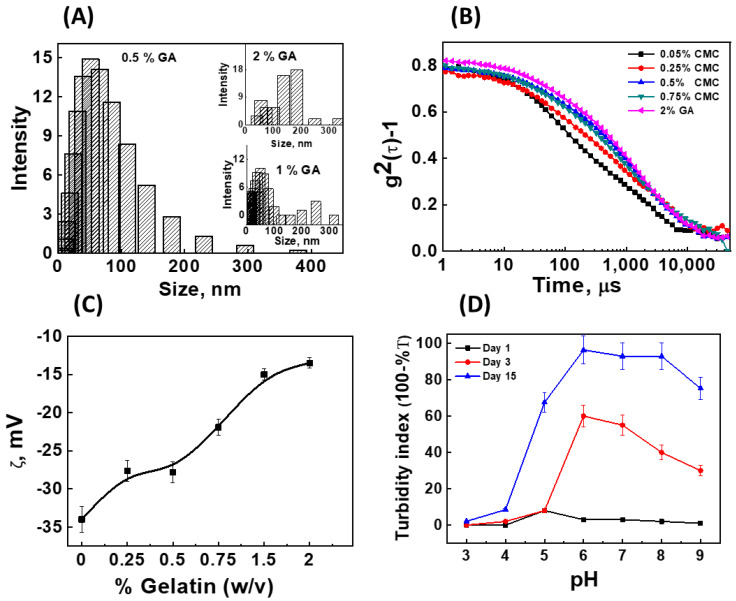
Plot (**A**) shows the intensity distribution of varying concentrations of GA. Plot (**B**) shows the correlation decay in the light scattering intensity of 2% GA in the absence and presence of different concentrations of CMC (0.05–0.75%). Plot (**C**) shows the variation in the zeta potential of the CMC solution when adding different amounts of GA (0–2%). Plot (**D**) shows how the turbidity index varies the pH of the solution.

The formation of composite colloidal particles or aggregates through the interaction between GA and CMC was studied via the employment of light scattering techniques such as DLS and zeta (ζ)-potential. Initially, the effect of the GA concentration on its own hydrodynamic size was examined. The aqueous solution (pH = 7) of GA showed a concentration-dependent increase in its own hydrodynamic size. For instance, at a concentration of 0.5% (*w*/*v*), GA showed a hydrodynamic size of 52 ± 4 nm (PI = 0.1), which increased to 65 ± 6 nm (PI = 0.1) and 184 ± 75 nm (PI = 0.4) after increasing the GA concentration to 1% (*w*/*v*) and 2% (*w*/*v*), respectively (Figure 1A). The size of the GA at a higher concentration (>2% *w*/*v*) could not be estimated through DLS as the auto-correlation function of such solutions failed to show the single relaxation decay. Instead, the decay profile of such concentrated solutions exhibited a biphasic mode comprising the faster and slower decay components, which are assigned due to the collective motion of the entangled chains and the translational diffusion of the GA cluster [38]. Therefore, all the subsequent experiments were performed while the GA concentration was restricted to 2% (*w*/*v*). The blank CMC (up to 2% *w*/*v* in water, pH = 7) did not show any characteristic correlation decay in the time scale of the DLS experiment. The addition of 0.01% (*w*/*v*) CMC to an aqueous (pH = 7) solution of 2% (*w*/*v*) GA resulted in a decrease in the hydrodynamic size of the mixture to 113 ± 21 nm (PI = 0.2), compared to the pure GA solution (184 ± 75 nm, PI = 0.4). Such a decrease in the size is evidence of the interaction between GA and CMC and the formation of the composite particles (Figure 1B). The effect of the complexation was further studied by varying the concentration of CMC from 0.05% to 0.75% in the presence of 2% GA (Figure 1B). The addition of 0.05% CMC also showed a decrease in the size of the nanocomposites to 75 ± 18 nm (PI = 0.3). The further increase in the concentration of CMC resulted in a gradual increase in the size of the nanocomposites, and this effect was saturated at a 0.5% (184 ± 17 nm, PI = 0.1) concentration of CMC. Therefore, from these studies, it was inferred that the weight ratio of 4:1 of GA to CMC (2% *w*/*v* of GA and 0.5% *w*/*v* of CMC in water) was the optimal ratio to form the colloidal particles (in terms of narrow size distribution). The nature of the complexation between the macromolecules was confirmed by monitoring the change in the zeta potential of CMC in the presence of different concentrations of GA. The 0.5% (*w*/*v*) aqueous solution (pH = 7) of CMC showed a zeta potential of −34.0 mV. The negative charge on CMC at neutral pH levels is attributed to the presence of the free carboxylate functional group. Furthermore, on the addition of increasing the concentration of GA, the zeta potential of nanocomposites presented a gradual decrease. Notably, in the presence of 2% (*w*/*v*) GA, the zeta potential value of nanocomposites decreased to −13 mV (Figure 1C). The phase plot of the complex demonstrated the formation of a single species with a lower zeta potential [39]. The decrease in the surface charge of CMC in the presence of GA also confirmed that the interaction between the macro-molecule was predominantly electrostatic in nature. Due to the existence of the electrostatic interface between CMC and GA, the extent of the interaction between them and the growth of the resulting colloidal particles can be tuned by modulating the net charge on the respective macromolecules, and by aging the reaction mixture for a longer time period, respectively. The net charge on the macromolecules can be modulated by changing the pH of the solution. Therefore, the complexation behavior of the 2% (*w*/*v*) GA with 0.5% (*w*/*v*) CMC in the aqueous solution was studied at varying pH levels, from 3 to 9, and aging the samples up to 15 days at room temperature. The change in the physicochemical properties was examined by studying the change in the colloidal turbidity on days 1 (24 h of aging or equilibration), 3, and 15. Visually, the CMC-GA complex precipitated immediately at pH levels of 3 and 4, whereas the CMC-GA complex existed as a stable colloidal system in the pH range from 5 to 9 at all the studied time points. The turbidity measurement is an indirect and convenient technique for extrapolating the size of a colloidal system. As seen in Figure 1D, the turbidity index of the CMC-GA complex at pH 5–9 increased with time, suggesting an increase in the size of colloidal particles. The complex prepared at pH 6–7 showed the highest turbidity index at the end of study period (day 15). The effect of the pH on the size of the composite colloidal particle on day 1 was also investigated through DLS. As the samples at pH levels of 3 and 4 were precipitated, they were excluded from the DLS studies. As seen in Figure S2, the relaxation time of the autocorrelation function of the complexes of day 1 decreased with the increase in the pH, and, from the fitting of the autocorrelation function, the hydrodynamic size of the particles at pH 5, pH 7, and pH 9 were estimated as 4916 ± 2120 nm, 582 ± 47 nm, and 487 ± 52 nm, respectively. The particle size of the CMC-GA complexes aged for longer time points (day 3 and 15) could not be examined through DLS measurements; this was due to their larger size (µm range), as supported by the turbidity data. The solubility and the surface charge of CMC in the acidic medium is low, and this accounts for the precipitation or the larger particle size of the complex in the pH range of 3–5. On the other hand, CMC has isoelectric points (IEP) at a pH of 6; therefore, CMC is expected to have varying extents of deprotonation of its carboxylic group, with a net negative charge at pH ≥ 6. This, in turn, is believed to favor the formation of stable colloidal systems with a tunable size. Together, pH studies revealed that the strong complexation between GA and CMC could be achieved by adjusting the pH of the reaction mixture from 5 to 9, and allowing the solution to equilibrate for a longer duration. Earlier studies have indicated that cosmetic formulations should have larger particle sizes (100 nm to few µm) in order to qualify for the green standard [40]. Taking this into account, it was inferred that the colloidal particles obtained through the incubation of the CMC-GA mixture for up to 3 days would be desirable as an emulsifier. Accordingly, in all the subsequent studies, 3 days of incubation was fixed to prepare the CMC-GA particle as the emulsifier. 

### 3.2. Exploring Composite Colloidal Particles as Emulsifiers for Coconut Oil and Water

**Figure 2:**
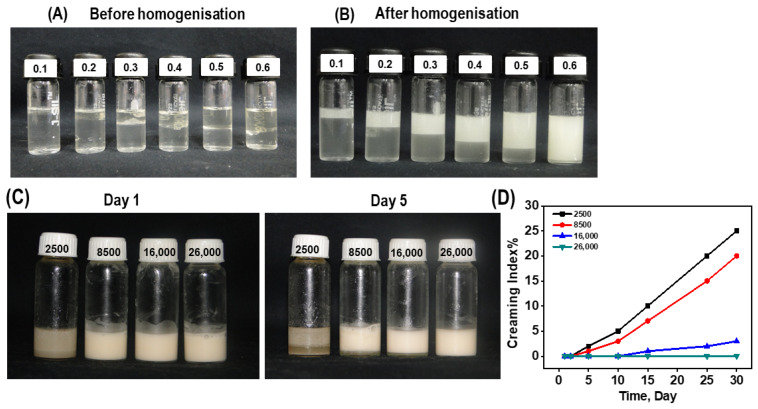
Photographs of the colloidal particles before (**A**) and after (**B**) homogenization with different (0.1–0.6) weight fractions of coconut oil. Plot (**C**) shows photographs of the emulsion prepared from 0.6 volume fraction coconut oil, under varying homogenization speeds (2500–26,000 rpm) on day 1 and day 5. Plot (**D**) shows the rates of the formation of the creamy layer in the emulsion prepared at different homogenization speeds.

After the optimization of the method to produce stable composite colloidal particles, their ability to emulsify coconut oil into water was investigated. Firstly, the ability of the composite colloidal particles to emulsify the volume fraction of coconut oil was optimized. For this, GA-CMC composite particles (2% *w*/*v* of GA and 0.5% *w*/*v* of CMC in water) were prepared at a pH of 7 and aged for 3 days, then mixed with the varying volume fractions (0.1 to 0.7) of coconut oil, and homogenized at a fixed speed of 16,000 rpm. Figure 2A,B and Figure S3 show the respective photos of the homogeneity of the emulsions at different volume fractions of coconut oil, both before and after the homogenization. The migration of the dispersed phase under the influence of buoyancy results in phase separation, which is undesirable for the stability of an emulsion. The emulsions prepared at volume fractions between 0.1 to 0.5 and of 0.7 of the coconut oil were not stable as the phase separation into cream and serum could clearly be seen in the pictures.

**Figure 3:**
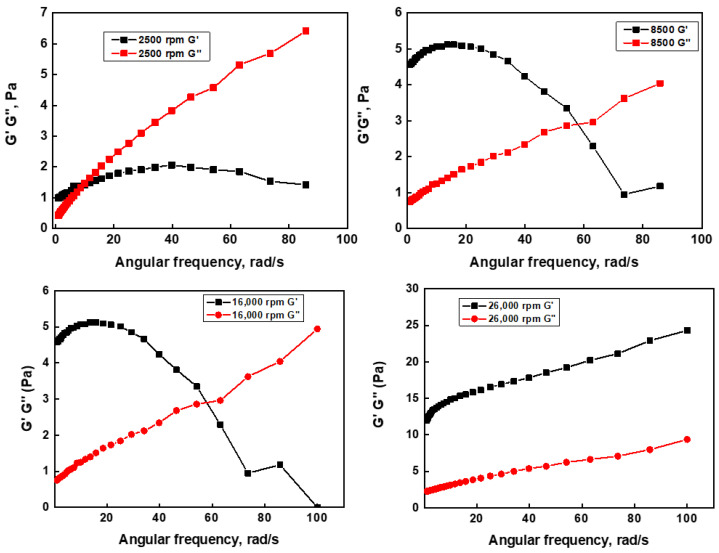
Rheological parameters (G′ and G) of the emulsion formed at different rpm.

The emulsions prepared at a volume fraction of 0.6 did not show any phase separation into cream and serum, suggesting higher levels of stability. Therefore, it was inferred that the 0.6 volume fraction of coconut oil was ideal for the preparation of the stable emulsion. The strength of the sheer force applied during the preparation of the emulsion also plays a significant role in its stability. Therefore, to study the effect of the speed of homogenization on the stability of th Pickering emulsion, the volume fraction of coconut oil in the water was fixed at 0.6, and the speed of homogenization varied from 2500 rpm to 26,000 rpm. Figure 2C shows the respective photos of the homogeneity of the emulsion at varying homogenization speeds (2500–26,000 rpm) on the first and the fifth day following synthesis. Visually, it can be inferred that the speed of homogenization had a strong influence on the stability of the emulsion. The emulsions prepared at 2500 to 8500 rpm were not stable until the fifth day, as the phase separation into cream and serum could be clearly seen on the fifth day, whereas the emulsion prepared at 16,000–26,000 rpm showed little to no phase separation on the fifth day. Supporting this, Figure 2D shows the creaming index of emulsions at different homogenization speeds as a function of time (30 days). It can be clearly seen that the creaming index of the emulsions prepared at 2500 to 8500 rpm increased as the days passed. On the other hand, emulsions prepared at 16,000 to 26,000 rpm did not show any significant increase in the creaming index after up to 30 days of preparation, suggesting better stability. Furthermore, to corroborate the above results, the stability of the as-synthesized Pickering emulsions was also monitored in terms of viscoelastic parameters, such as G′ and G″, through the rheometric studies [41,42]. In order to determine the G′ and G″, the emulsions were subjected to deformation in a frequency sweep at a given strain of 1%. The intersection or crossover of G′ and G″ is indicative of the instability of the gel [41,42]. The dynamic frequency sweep test indicated that the emulsions prepared at 2500, 8500, and 16,000 rpm exhibited G′ > G″ at lower frequencies, and the cross over at higher frequencies (Figure 3). 

**Figure 4:**
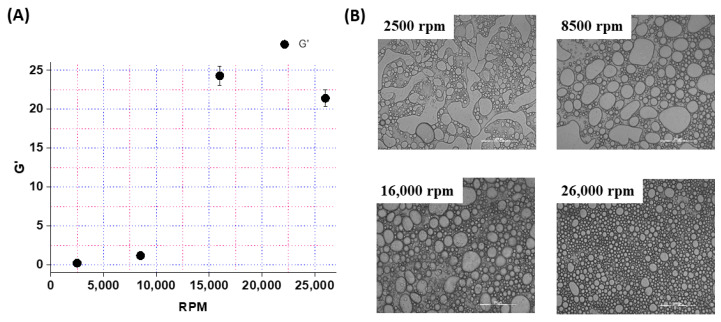
(**A**) Plot of storage modulus (G′) at the start of the frequency sweep against the homogenization speed. Plot (**B**) shows the optical images of the emulsion microstructure obtained at different speeds of homogenization, as observed under the microscope (Magnification 10×).

However, the emulsion prepared at 26,000 rpm showed parallel changes in the values of G′ and G″ throughout the applied strain. Furthermore, the loss factor or tan (δ) (expressed as the ratio of G″ and G′) for the emulsion prepared at 2500 rpm was close to 1 (G″ = G′) at a lower frequency, whereas the emulsions prepared at 8500, 16,000, and 26,000 rpm showed tan δ < 1 (G″ < G′) at lower frequencies. A value of tan δ < 1 (G″ < G′) suggests the elastic nature of material, whereas a value of tan δ > 1 (G″ > G′) suggests the viscous nature of the material [42]. Thus, the above results suggested that emulsions prepared at higher homogenization speeds were elastic in nature with a better stability or gel strength (G′) than those prepared at low homogenization speeds. Indeed, the plot of G′ versus the homogenization speed showed a sigmoidal behavior, with an inflection point at 16,000 rpm (Figure 4A). Thus, a minimum shearing stress was required for the homogenization or the breaking down of the oil droplets into a stable emulsion. In order to confirm this, the size distribution of the oil droplets in the as-synthesized Pickering emulsions was characterized through optical microscopy. As seen in Figure 4B, the oil droplets of the emulsions prepared at 2500 rpm and 8500 rpm were quite large and polydispersed, whereas the droplet size decreased with the increase in the homogenization speed from 16,000 to 26,000 rpm. For instance, the droplet size at 2500 rpm was found to be 1000–2000 µm, which decreased to 50–100 µm at 26,000 rpm. The smaller droplet size is indicative of the higher stability of the emulsion. Additionally, from the comparison of the optical image of the elusions at different rpm, it can be inferred that the oil droplets are closely packed at a higher rpm when compared to the lower rpm value. In line with the above observation, the emulsions prepared at 16,000 and 26,000 rpm showed a higher viscosity of 100–120 cP, whereas the emulsions prepared at 2500 and 8500 exhibited a low viscosity in the range of 20–40 cP (Figure S4). Viscosity is the measure of a fluid’s internal flow resistance. Higher viscosity values indicate a larger interaction between the oil droplets and the colloidal particles within the emulsion. On the other hand, a lower viscosity indicates Newtonian fluid-like behavior, which may be attributed to the poor stabilization of the oil droplets, due to their larger size observed through the optical microscopy. Together, the 0.6 volume fraction of coconut oil and a homogenization speed of 16,000 rpm appeared to be the ideal conditions for the preparation of a CMC-GA-stabilized O/W Pickering emulsion. 

### 3.3. Effect of pH on the Stability of the CMC-GA-Emulsified Pickering Emulsion

**Figure 5:**
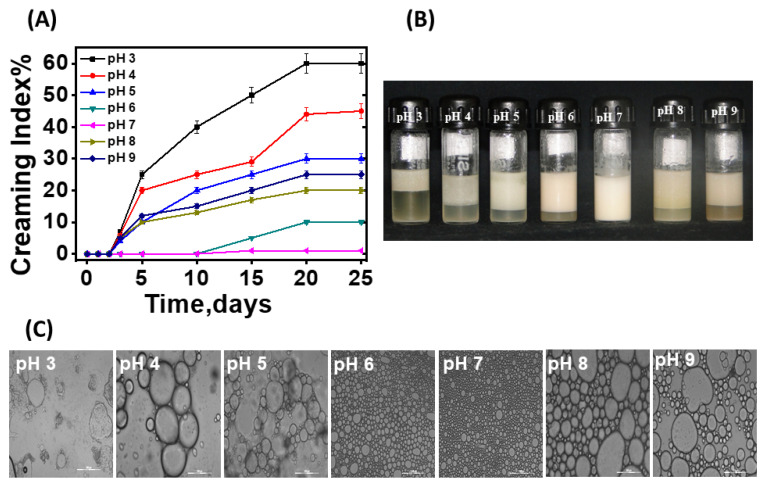
(**A**) Plot shows the rate of the formation of the creamy layer in the emulsion prepared at different pHs. (**B**) Photographs of the emulsions prepared at different pHs on day 5. (**C**) Optical images of the emulsion microstructure, obtained at different pHs as observed under a microscope (Magnification 10×).

Following the optimization of the oil volume fraction and the homogenization speed, it was important to understand whether CMC-GA particles of varying pH levels can yield a stable Pickering emulsion. In order to address this, CMC-GA colloidal particles of varying pH levels (3–9) were used to emulsify coconut oil in water, using previously optimized conditions (an oil volume fraction of 0.6 and a homogenization speed of 16,000 rpm). The stability of the resulting emulsion was monitored by assessing the phase separation, oil droplet size, and viscosity. Figure 5A shows the plot of the creaming index of emulsions of varying pHs, up to 25 days from the preparation. It can be clearly seen that, except at a pH of 7, emulsions at all the other investigated pHs (3–6, 8 and 9) showed increases in the creaming index as a function of time. Furthermore, the creaming index at each time point decreased from pH 3 to pH 6, and again increased from pH 8 to pH 9. Notably, the emulsion at pH 7 did not show any increase in the creaming index after up to 25 days, suggesting the highest stability. The respective photos of the emulsions of varying pHs on the first and the fifth days following preparation also support the above observation. For instance, except at pH 7, emulsions at all the other investigated pHs (3–6, 8 and 9) showed phase separation by the fifth day (Figure 5B). Furthermore, the optical microscopy of the emulsions showed that the oil droplets of the emulsions were quite large, and polydispersed at pHs of 3 and 4. The droplet size of the emulsions decreased with an increase in the pH up to 7, whereas the subsequent increase in the pH (>7) of the emulsions showed increases in the droplet size (Figure 5C). Finally, viscosity measurements indicated that the viscosity of the emulsion increased from 20 cP at pH 3 to 130 cP at pH 6–7 (Figure S5A). A higher increase in the pH resulted in a decrease in the viscosity of the emulsion. Together, the above studies indicate that CMC-GA colloidal particles of pH 7 were ideal for the preparation of stable Pickering emulsions. In order to understand the reason for the above observations, the emulsions at different pHs were characterized by the surface charge. As seen in Figure S5B, the surface charge of the emulsion was positive at acidic pHs, and the value tended to lean towards the negative surface charge with an increase in the pH. The sigmoidal curve of the surface charge against the pH showed an inflection point at a pH around 5.8. At this pH, the zeta potential of the colloidal system was nearly zero. This indicated that the stabilization of the oil at the interface required a near-zero surface charge on colloidal particles, and also likely involved hydrophobic forces.

### 3.4. Loading of Curcumin and Estimating the Sun Protection Factor (spf)

**Figure 6:**
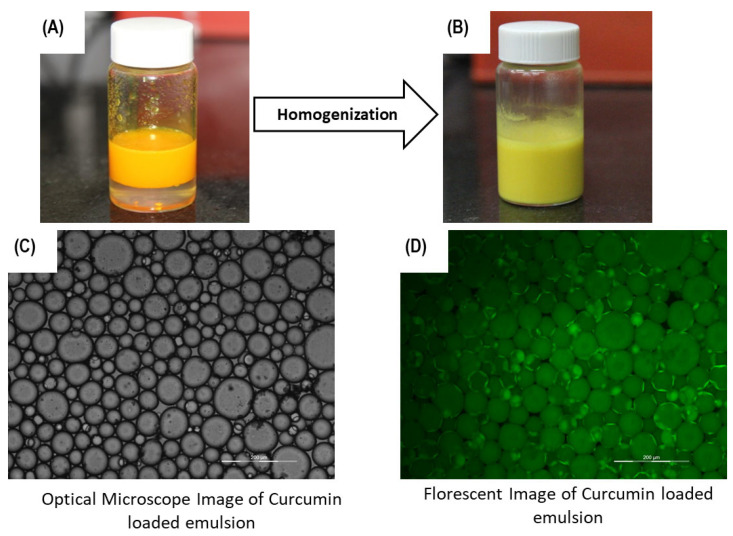
Photographs of curcumin loaded in the emulsion (**A**) before and (**B**) after homogenization. Images (**C**,**D**) represent the image of the curcumin-loaded emulsion under (**C**) an optical microscope and (**D**) a fluorscence microscope (Magnification 20×).

Next, the loading of curcumin into the Pickering emulsion was studied. For this, curcumin dissolved in the 0.6 volume fraction of coconut oil was mixed with CMC-GA composite colloidal particles, as optimized in the previous sections, and homogenized at 16,000 rpm. The encapsulation efficiency of curcumin at final concentrations of 2 mg/mL and 4 mg/mL was estimated as 99% and 97%, respectively (Figure S6A,B). The microstructure and loading of curcumin were confirmed by bright field and fluorescence imaging, respectively. As seen from Figure 6 and Figure S7, the Pickering emulsion could easily load the curcumin to form a yellow-colored emulsion. The final curcumin-loaded formulation showed an oil droplet size of 100 ± 20 μm. Thus, using this Pickering emulsion, curcumin up to 4 mg/mL was solubilized with minimum precipitation. Furthermore, curcumin is widely used in sunscreen cream formulations. Therefore, the SPF of the curcumin-loaded Pickering emulsion was determined as per the method section. The SPF value of the formulation at a curcumin loading of 4 mg/mL was calculated as 8.5.

### 3.5. Estimation of the Cellular Uptake of Curcumin through the Pickering Emulsion

**Figure 7:**
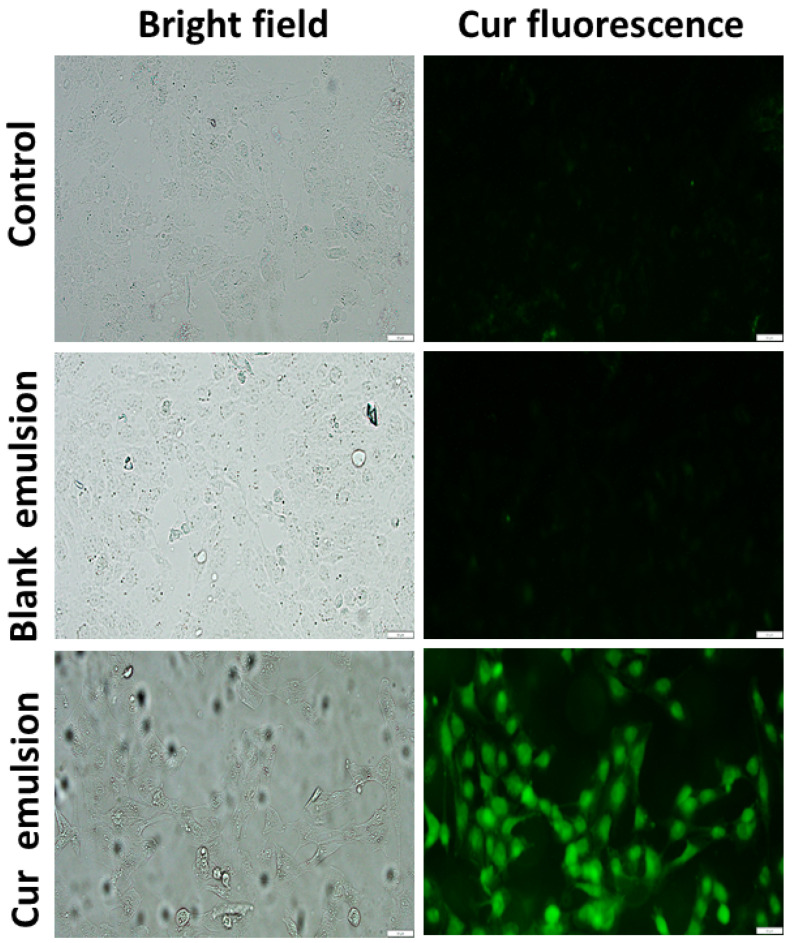
The figure shows the representative bright field and fluorescence images of WI26 cells treated with 25 µM curcumin equivalent of blank and curcumin-loaded GA-CMC stabilized Pickering emulsions. The cells were treated with the formulations for 6 h. The fluorescence images were captured using the IX83 (Olympus) microscope following excitation, using a band pass fluorescence filter set of FITC. Control represents untreated cells. The magnification was 20×.

Though cellular uptake is not an important parameter for sun screening agents, it may be advantageous in the case of curcumin-based cosmetic formulations. Intracellular curcumin can also participate in preventing phototoxicity, as well as in general skin care [2,3]. Therefore, the uptake of curcumin from the coconut oil–water Pickering emulsion into normal human fibroblast (WI26) cells was studied. For this, the cells were treated with the equivalent of 25 µM of curcumin in the coconut oil-water Pickering emulsion, and the amount of the curcumin uptake was estimated quantitatively by extracting curcumin from the cell lysate and qualitatively through fluorescence imaging, as mentioned in the method section. Notably, the amount of curcumin estimated in the cells treated with 25 µM curcumin equivalent of the coconut oil-water Pickering emulsion was 4.40 ± 0.03 nmoles curcumin/mg of protein. It can also be argued that the cellular uptake determined by the above method may include contributions from the membrane-bound curcumin, and this should not be considered cellular uptake. In order to address this concern, the cellular uptake of curcumin through the CMC-GA-stabilized Pickering emulsion was also evaluated through fluorescence microscopy. As shown in Figure 7, the cells treated with the blank Pickering emulsion did not show any fluorescence upon excitation. However, the cells treated with the curcumin-loaded coconut oil–water Pickering emulsion showed bright fluorescence upon excitation. Notably, the fluorescence intensity was localized mainly inside the cells, confirming the cellular uptake of curcumin. The bright field images of the cells treated with the blank Pickering emulsions showed intact morphology, suggesting the non-toxic nature of the formulation. Taken together, the CMC-GA-stabilized Pickering emulsion of curcumin is expected to participate in skin care through the extracellular absorption of solar radiations, as well as through intracellular protection due to the curcumin uptake. 

## 4. Discussion

There are several reports available in the literature on the entrapment of curcumin in the classical emulsions, as well as in the Pickering emulsions [10,43,44,45,46,47]. These studies have mostly focused on the efficiency of the emulsions as a delivery vehicle for the release of curcumin into cells/tissues, which includes its dermal applications. For instance, Scomoroscenco et al. has recently reported the development of a novel gel-based microemulsion for the topical delivery of curcumin [10]. Briefly, the emulsion was fabricated using a mixture of Tween 80 and Plurol^®^ Diisostearique CG as the emulsifier, and grape seed oil as the oily phase. To impart the gelling characteristics, three different water-soluble polymers viz., Carbopol^®^ 980 NF, chitosan, and sodium hyaluronate salt, were added into the emulsion. The fabricated gel microemulsion exhibited a controlled release and the dermal penetration of curcumin under in vitro conditions. In another study, Juan Luis Pérez-Salas et al. studied the effect of the concentration of polyglycerol polyricinoleate (PGPR) on the fabrication of W/O gelled emulsions, and evaluated the mechanisms for the entrapment and release of curcumin [43]. The results indicated that a higher concentration of PGPR reduced the affinity between curcumin and the emulsion, thereby facilitating the release of curcumin through the Fickian mechanism. Furthermore, Ergin et al. has reported the fabrication of a surfactant-free emulgel formulation of curcumin through the use of Carbopol 940 gels as the aqueous phase, and the use of an olive oil and curcumin methanol solution as the oily phase [44]. The obtained emulgels showed good mechanical, rheological, and spreadability properties, and also increased the transdermal permeation of curcumin. Regarding the Pickering emulsion, curcumin has been entrapped into O/W Pickering emulsions, which were stabilized using a variety of surfactant-free colloidal particles, such as electrostatically assembled otransferrin–gallic acid conjugates (OTGCONJ) and carboxymethyldextran (CMD) particles, starch–chitin nanoparticles, zein–propylene glycol alginate (PGA)-tea saponin (TS) complex nanoparticles, and several other diverse biopolymeric particles [45,46,47]. All such formulations have shown improvements in the chemical stability and bio-accessibility of curcumin under in vitro and in vivo conditions. However, to the best of our knowledge, there are no reports available on the preparation of curcumin-loaded Pickering emulsions for cosmetic applications. The formulations intended for cosmetic application mainly act as shielding materials on the surface of the skin, used to absorb the solar radiation [18,29,30,40]. Moreover, with the growing evidences, it has emerged that the presence of antioxidant/anti-inflammatory ingredients in the sun screening formulations further enhances their effectiveness by preventing the solar radiation-induced intracellular oxidative damage via biological mechanisms [2,3,9]. With this background, the present investigation explores the potential of CMC-GA composite colloidal particles as emulsifiers for the preparation of curcumin-loaded water–coconut oil Pickering emulsions and its possible application as a green cosmetic formulation. The novelty of the present study comes from the choice of GA, CMC, and coconut oil as the ingredients of the Pickering emulsion, as these reagents are biodegradable and already the constituents of several approved cosmetic formulations [30,48,49]. Furthermore, curcumin entrapped within the emulsion can also contribute to the sun screening properties via not only the absorption of solar radiation in the UV range of 420 nm, but by preventing solar radiation-induced intracellular damage through antioxidant mechanisms [2,3]. Moreover, all the excipients used in the present study, such as GA, CMC, curcumin, and coconut oil, are of a pharmaceutical grade, and are used well within their reported toxicity limits, thus ensuring the green nature of the as-prepared formulation [50,51,52]. 

With regard to the mechanism of the preparation of the Pickering emulsion, GA and CMC are polyelectrolytes with an IEP of 8.5 and 5.5, respectively [53,54]. Accordingly, these biopolymers are expected to have positively and negatively charged surfaces at a neutral pH (~7), and this allowed the complexation between them through the electrostatic interaction to form the composite particles with a narrow size distribution. The concentrations of the GA and CMC required for stable complexation are optimized as a 4:1 weight ratio. These results appear encouraging, as emulsifiers intended for cosmetic applications are expected to be stable at physiological pHs (~7). Subsequently, the Pickering emulsion of coconut oil in water is prepared using CMC-GA composite particles as the emulsifier. The detailed analysis of the stability of the as-synthesized Picketing emulsion indicated that CMC-GA composite particles showed optimum emulsifying properties at a 0.6 volume fraction of oil and a homogenization speed of 16,000 rpm. The emulsion prepared under the above conditions showed a smaller size of oil droplets, a higher mechanical strength (G′) and viscosity, and a lesser phase of separation into cream and serum. All these parameters are suggestive of a better stability of the Pickering emulsion. Previously, Wang et al. have synthesized a Pickering emulsion of corn oil in water by using an insoluble complex, derived from chitosan and gelatin B [54]. The present finding is consistent with the above report, where increasing the homogenization speed resulted in the breakdown of the larger droplets. However, the size of the droplets obtained in the present investigation is smaller, suggesting a better emulsification than the previous system. Since the net zeta potential values of the composite CMC-GA particles at a neutral pH are about −13 mV (which is close to a neutral surface charge) [38], it is inferred that the hydrophobic interaction is the major stabilizing force between the composite CMC-GA particles and coconut oil droplets. Similarly, Wang et al. have also reported that gelatin B–chitosan complex Pickering emulsions are more stable at a neutral pH where the zeta potential value is nearly zero [55]. Finally, curcumin loading of 4 mg/mL was achieved in coconut oil–water Pickering emulsions, which is the highest loading capacity from any surfactant-free delivery system [47,48,56,57]. Remarkably, the curcumin-loaded Pickering emulsion exhibited a SPF well within the range required for sun screen formulations [18,35]. Additionally, the formulation also showed the release of curcumin into a model cellular system of fibroblast origin. The skin fibroblast cells are generally used as cellular models to estimate the cellular uptake of formulations intended for topical application. However, due to the unavailability of skin fibroblast cells, we employed lung fibroblast cells. Nevertheless, the cellular uptake study suggests that the absorbed curcumin can also participant in skin care through its well-known antioxidant and anti-inflammatory mechanisms [2,3]. All these observations warrant future in vivo investigations exploring various skin-care applications, such as the sunscreen, cosmetic, the wound-healing properties of the newly designed CMC-GA-stabilized Pickering emulsion containing curcumin. 

## 5. Conclusions

Briefly, the methodologies to prepare the electrostatically complexed composite colloidal particles comprising CMC and GA, and their subsequent applications as an emulsifier to prepare curcumin-loaded Pickering emulsions have been optimized. The results demonstrate that the ability of CMC-GA particles as an emulsifier to form the Pickering emulsion is greatly affected by pH, the volume of oil fraction, the homogenization speed, and storage time. In vitro studies also confirm that curcumin-based Pickering emulsions have reasonable SPF levels and are also capable of delivering curcumin to cells.

## Data Availability

All data are contained within the article.

## Supplementary Materials

The following supporting information can be downloaded at https://www.mdpi.com/article/10.3390/pharmaceutics16030356/s1, Figure S1: Estimation of pKa in (A) CMC, (B) GA, and (C) the conductimetric titration of CMC to estimate the degree of deacetylation; Figure S2: Variation of electric field correlation function (G1(τ)) of composite colloidal composites obtained at different pHs. The intensity correlation function was analyzed through the method of cumulants; Figure S3: Photographs of the GA-CMC colloidal particles after homogenization with different (0.1–0.7) weight fraction of coconut oil. The arrow indicates the phase separation between cream and serum. Figure S4: Variation in the viscosity of the Pickering emulsion formed at different homogenization speeds (■ = 2500 rpm, ● = 8500 rpm, ▲ = 16,000 rpm and ▼ = 26,000 rpm); Figure S5: (A) Variation in the viscosity of the Pickering emulsion formed at different pHs. (B) Plot shows the variation in the zeta potential of the emulsion as a function of pH. Figure S6: Graph (A) shows the absorption spectrum of curcumin obtained on diluting 10 μL of curcumin-loaded Pickering emulsions in 3 mL acetonitrile solvent. Graph (B) shows the plot of absorbance against curcumin concentration. Here, the slope of the linear fit equates to the extinction coefficient of curcumin in acetonitrile solvent at 420 nm; Figure S7: Confocal images of curcumin-loaded Pickering emulsions at different magnifications (10×, 20×, 63×). The last two images (right side) on the lower panel represent 3D views obtained through Z stacking.
