# Supplementary material for: The Preparation of Curcumin-Loaded Pickering Emulsion Using Gelatin–Chitosan Colloidal Particles as Emulsifier for Possible Application as a Bio-Inspired Cosmetic Formulation
Source: Pharmaceutics. 2024 Mar 3;16(3):356. doi: 10.3390/pharmaceutics16030356 (PMC10975880; doi:10.3390/pharmaceutics16030356)
Supplement: Supplementary file 1 [file pharmaceutics-16-00356-s001.zip › pharmaceutics-2889903-supplementary.pdf]

# Supporting Information

# The Preparation of Curcumin-Loaded Pickering Emulsion Using Gelatin–Chitosan Colloidal Particles as Emulsifier for Possible Application as a Bio-Inspired Cosmetic Formulation

**Beena G. Singh [1,2,†], Nalin Bagora [3,†], Minati Nayak [1,2], Juby K. Ajish [1] and Nitish Gupta [3] and Amit Kunwar [1,2,*]**

[1] Radiation and Photochemistry Division, Bhabha Atomic Research Centre, Trombay, Mumbai 400085, India; beenam@barc.gov.in (B.G.S.); nminati@barc.gov.in (M.N.); kuttan@barc.gov.in (J.K.A.)
[2] Homi Bhabha National Institute, Anushaktinagar, Mumbai 400094, India
[3] Department of Applied Chemistry, S. G. S. Institute of Technology and Science, Indore 452003, India; bagoranalin1995@gmail.com (N.B.); nitish.nidhi75@gmail.com (N.G.)
* Correspondence: kamit@barc.gov.in
† These authors contributed equally to this work.

***Supplementary methods***

*1. Characterization of polymer*

*1.1 Estimation of isoelectric point of the macromolecules*

Due to the presence of functional groups, the structural transition of GA is influenced by pH. At the isoelectric point (IEP), GA is globular in shape and has a smaller hydrodynamic radius and low viscosity. When the pH is different from the IEP, GA has either more positive or negative charges, resulting in a linear structure, with a larger hydrodynamic radius and high viscosity. Similarly, it is reported that chitosan exhibits pH dependent structural changes. Due to this, it is important to estimate the IEP of the macromolecule. The pH of the aqueous solution of 0.5% CMC was found to be 8.64, which may be due to the presence of sodium salt of CMC. To estimate the isoelectric point, the pH of the solution was adjusted to 9 and further titrated with 0.1 N HCl. As seen from figures S1A and S1B, the inflection points which corresponds to the IEP of CMC and GA were estimated as 5.9 and 7.9 respectively.

*1.2 Degree of deacetylation of CMC*

Chitosan is composed of randomly distributed D-glucosamine and its acetylated derivative - N-acetyl-D-glucosamine. The D-glucosamine content which is expressed as degree of deacetylation (DD) is found to influence the physical, chemical and biological properties of chitosan. This is also a useful parameter to differentiate between chitin and chitosan. The average degree of deaceytlation in chitosan ranges between 60-90%, whereas in chitin, it is in the range of less than 17%. In the present study, the average degree of deacetylation (DDA) in chitosan sample was determined by employing conductimetric titration. On titration with 0.1 N NaOH in an increment of 50 μl to the solution, gave two inflection point as shown in figure S1C. Employing

the equations 1, DDA was estimated to be 80 %. This value is in agreement with the previous values reported in the literature [1,2].

The average degree of deacetylation (DDA) was calculated by using equation (1):

$$\% \text{ DDA } (NH_2) = 16 \times [y-x] / M \qquad (1)$$

Where M is the weight of CMC in 100 ml, x and y are the first and second inflection point in the graph.

Further, the DDA value obtained was employed to estimate the average molecular weight of CMC by using Mark-Houwink equation (equation 2). The equation uses the relationship between viscosity and molecular weight. The molecular weight obtained by this technique is called viscosity average molecular weight.

$$[\eta] = K x\ M\alpha \qquad (2)$$

Where [η] is the intrinsic viscosity, M is the molecular weight, and K and $\alpha$ are constants determined experimentally for a given polymer-solvent systems.

de Abreu et al related k and $\alpha$ values from DDA as shown in equation (3) and (4), respectively [1].

$$k = (1.64 \times 10\text{-}30) \times (DDA14.0)\ (mL/g) \qquad (3)$$

$$\alpha = (-1.02 \times 10\text{-}2) \times (DDA) + 1.82 \qquad (4)$$

The degree of deacetylation calculated by conductimetric titration was 80 %, giving value of $k = 1.959 \times 10^{-10}$ mL/g and $\alpha = 1.542$. The viscosity for CMC was determined to be 5.207. Thus using the Mark-Houwink equation the viscosity average molecular weight of the biopolymer was calculated to be a = 65,251 Dalton.

*References*

1. de Abreu, Fernanda R.; Campana, Sérgio P. Preparation and characterization of carboxymethylchitosan, Polímeros: Ciência e Tecnologia, 2005, 15, 79-83.

2. Haririan Y, Asefnejad A, Hamishehkar H, Farahpour MR. Carboxymethyl chitosan-gelatin-mesoporous silica nanoparticles containing Myrtus communis L. extract as a novel transparent film wound dressing. Int J Biol Macromol. 2023 Dec 31;253(Pt 5):127081. doi: 10.1016/j.ijbiomac.2023.127081. Epub 2023 Sep 26. PMID: 37769781.

*Supplementary Figures*

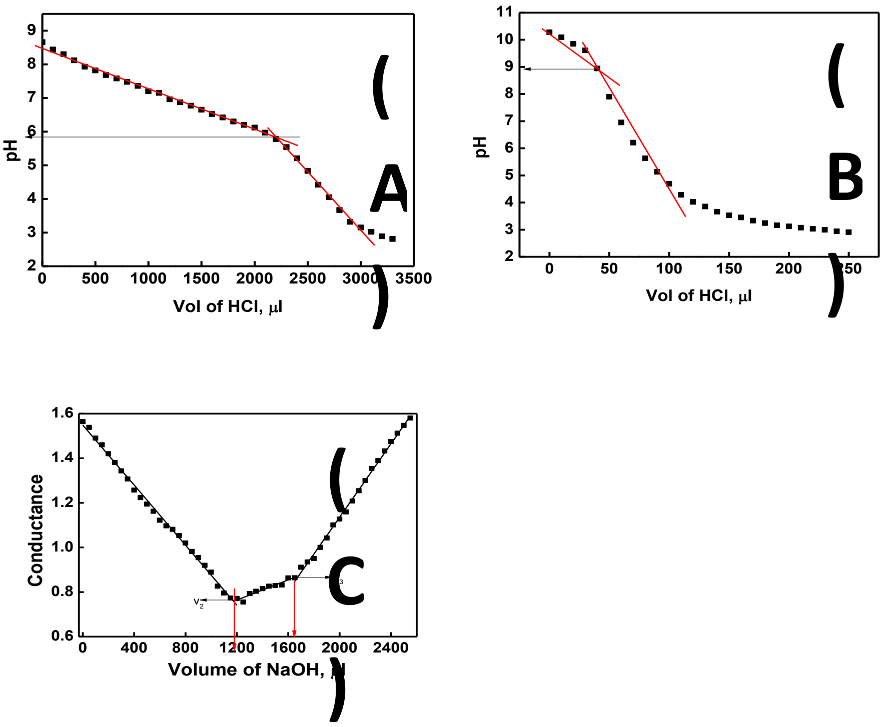


**Figure S1**: Estimation of pKa in (A) CMC, (B) GA and (C) Conductimetric titration of CMC to estimate the degree of deacetylation.

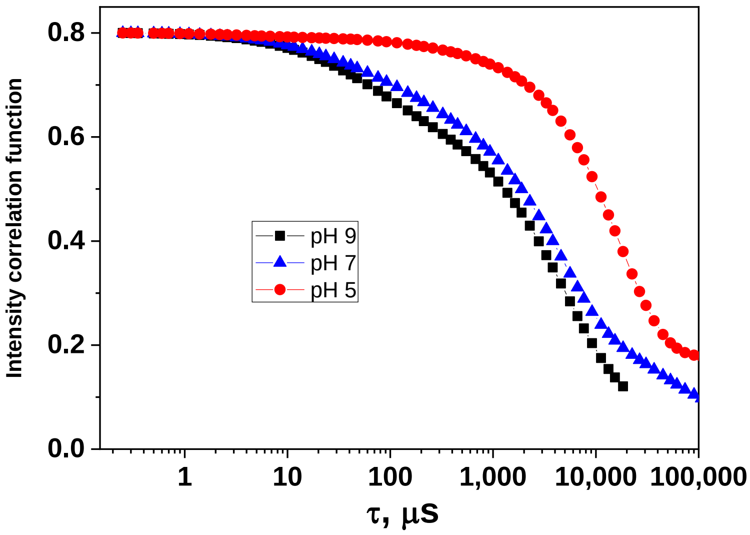


**Figure S2**: Variation of electric field correlation function (G1(τ)) of composite colloidal composite obtained at different pHs. The The intensity correlation function was analyzed by the method of cumulants.

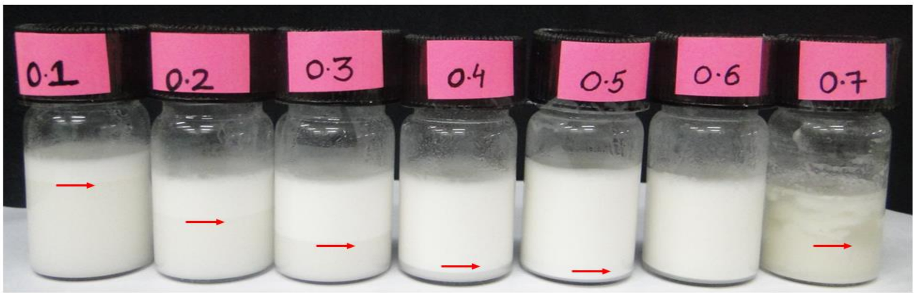


**Figure S3**. Photographs of the GA-CMC colloidal particles after homogenization with different (0.1 – 0.7) weight fraction of coconut oil. The arrow indicates the phase separation between cream and serum.

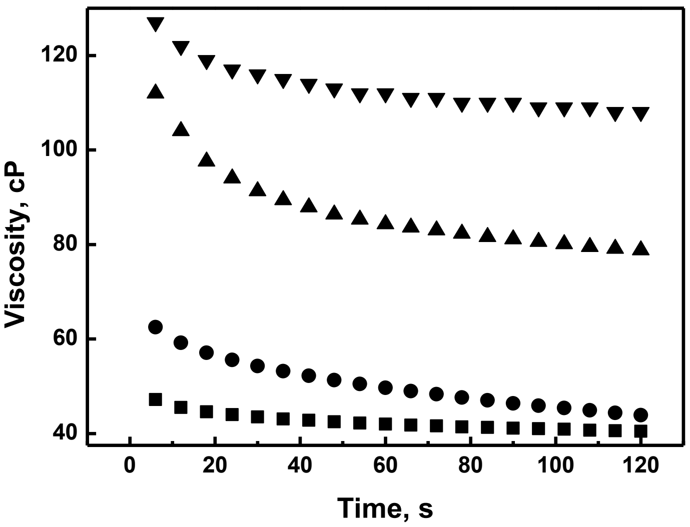


**Figure S4**: Variation in the viscosity of the Pickering emulsion formed at different homogenization speed (■ = 2500 rpm, ● = 8500 rpm, ▲ = 16000 rpm and ▼ = 26000 rpm).

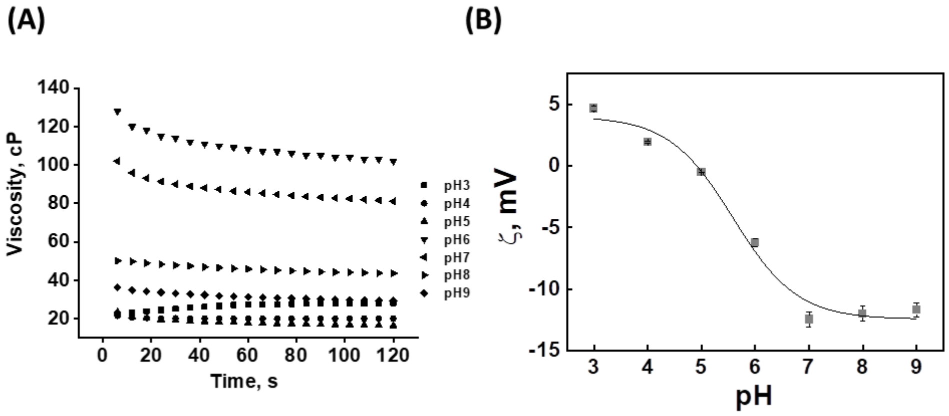


**Figure S5**: (A) Variation in the viscosity of the Pickering emulsion formed at different pHs. (B) Plot shows the variation in the zetapotential of the emulsion as a function of pH.

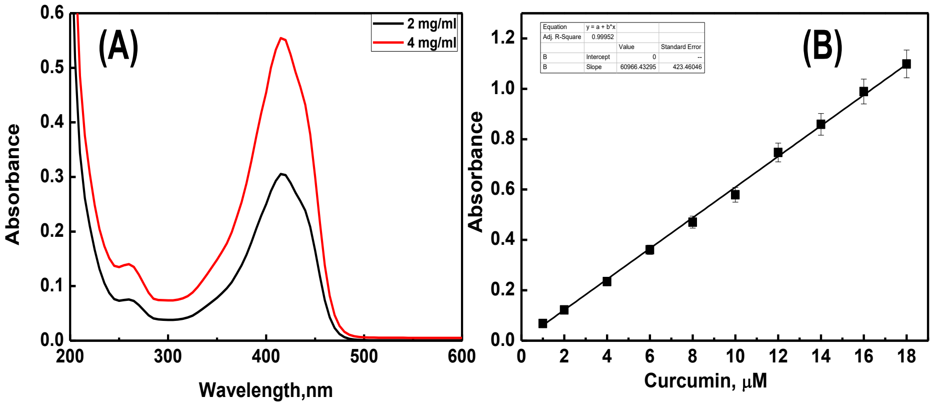


**Figure S6**: Graph (A) shows absorption spectrum of curcumin obtained on diluting 10 μl of curcumin loaded Pickering emulsion in 3 ml acetonitrile solvent. Graph (B) shows the plot of absorbance against curcumin concentration. Here the slope of the linear fit equates to the extinction coefficient of curcumin in acetonitrile solvent at 420 nm.

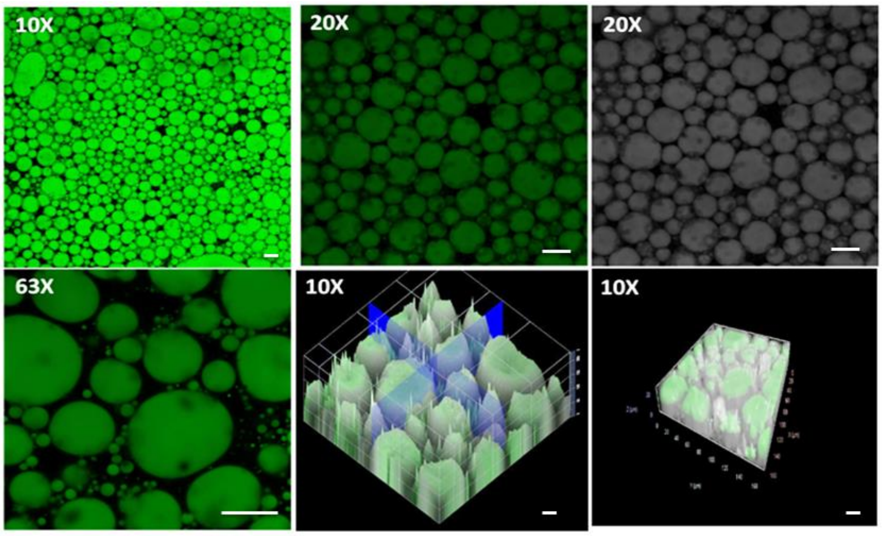


**Figure S7**: Confocal images of curcumin loaded Pickering emulsion at different magnification (10×, 20×, 63×). The last two images (right side) of lower panel represent 3D view ontained through Z stacking. Scale bar = 100 μm
